# Association of recipient and donor interleukin 6 polymorphisms 174 and 597 with outcome after allogeneic hematopoietic stem cell transplantation in children

**DOI:** 10.1007/s00432-021-03677-5

**Published:** 2021-06-12

**Authors:** Laura Wetzel, Susan Wittig, Bernd Gruhn

**Affiliations:** Department of Pediatrics, Jena University Hospital, Am Klinikum 1, 07747 Jena, Germany

**Keywords:** Interleukin 6, Single nucleotide polymorphism, Allogeneic hematopoietic stem cell transplantation, Children, Graft-versus-host disease

## Abstract

**Purpose:**

The success of allogeneic hematopoietic stem cell transplantation (HSCT) is compromised by complications such as infection, relapse, and graft-versus-host disease (GVHD). The investigation of non-HLA immunogenetics, particularly of cytokines, could identify predictors of an unfavorable outcome after allogeneic HSCT. In this study, we examined the impact of single nucleotide polymorphisms (SNPs) within the promoter region of interleukin 6 (IL6) on the development of GVHD after pediatric allogeneic HSCT.

**Methods:**

In this retrospective analysis, we included 320 pediatric patients with a median age of 10 years who underwent an allogeneic HSCT and their respective donors. We used TaqMan real-time polymerase chain reaction to analyze the SNPs IL6-174 (G/C) and IL6-597 (G/A). The IL6-174 polymorphism was examined in 300 recipients and 295 donors. The IL6-597 polymorphism was analyzed in 299 recipients and 296 donors. We investigated the influence of the IL6-174 and IL6-597 polymorphisms on overall survival, event-free survival, relapse incidence, transplant-related mortality, and the occurrence of GVHD.

**Results:**

G polymorphism at position 174 of the recipient IL6 gene was associated with a higher incidence of acute GVHD (GG vs. GC/CC; *P* = 0.024). Patients with IL6-597 GG genotype developed acute GVHD more frequently than individuals with an A allele (GG vs. GA vs. AA; *P* = 0.013). IL6-174 GG homozygous recipients had a more frequent occurrence of chronic GVHD (GG vs. GC/CC; *P* = 0.049). We observed a significant increased risk of chronic GVHD in recipients with IL6-597 GG genotype (GG vs. GA vs. AA; *P* = 0.043). Polymorphisms of donors did not affect the incidence of acute GVHD and chronic GVHD. In multivariate analysis, the IL6-174 and IL6-597 SNPs were independent significant risk factors for acute GVHD (*P* = 0.030; *P* = 0.007, respectively) as well as for chronic GVHD (*P* = 0.045; *P* = 0.015, respectively). In addition, older age at time of transplantation turned out to be a significant risk factor for chronic GVHD (*P* = 0.003).

**Conclusion:**

Our study identified the IL6-174 and IL6-597 GG genotypes of pediatric allogeneic HSCT recipients as genetic risk factors for the development of acute GVHD and chronic GVHD. After evaluations in further studies, these findings could implicate the adjustment of prophylactic measures to reduce the occurrence of acute GVHD and chronic GVHD.

## Introduction

Allogeneic hematopoietic stem cell transplantation (HSCT) is a curative treatment option for hematological malignancies, genetic diseases, and severe immune deficiencies. A variety of complications such as infection, relapse, and graft-versus-host disease (GVHD) influence morbidity and mortality after allogeneic HSCT (Ambruzova et al. 2008).

Acute GVHD and chronic GVHD are common and serious complications following allogeneic HSCT. Pro-inflammatory and anti-inflammatory cytokines play a crucial role in both diseases (Cavet et al. 2001; Dukat-Mazurek et al. 2017).

The development of acute GVHD is described as a three-phase process. The pretransplant conditioning regimen damages and activates the host tissue. The host cells produce cytokines such as interleukin 1, interleukin 6 (IL6), and tumor necrosis factor α and stimulate donor T cells. The activated T cells also release pro-inflammatory cytokines and reinforce the immune response. Finally, the recruited effector cells such as macrophages and natural killer cells cause the damage of the target organ (Reddy and Ferrara 2003). The pathophysiology of chronic GVHD is complex and characterized by immune dysregulation and absence of functional tolerance. Thymic damage, alloreactive as well as autoreactive B and T cells, and the mechanisms of chronic inflammation with subsequent fibrosis play a crucial role (Wolff and Lawitschka 2019). IL6 is an important mediator and regulator in acute and chronic GVHD. IL6 is a pleiotropic cytokine which forms an interface of adoptive and innate immunity (Muller-Steinhardt et al. 2009). It is produced by hematopoietic cells such as monocytes, macrophages, and T cells as well as by non-hematopoietic cells including adipocytes, endothelial cells, and fibroblasts (Marshall et al. 2001; Terry et al. 2000). It has both pro- and anti-inflammatory properties. IL6 stimulates hepatocytes to produce acute-phase proteins and contributes to the inflammatory reaction. Additionally, it has stimulating effects on B- and T-cell maturation and differentiation. IL6 inhibits the maturation of naive CD4 + T cells into regulatory T cells and promotes the differentiation of macrophages. IL6 stimulates T cells to produce interleukin 10 and controls the expression of interleukin 21 in T cells (Hunter and Jones 2017). IL6 is involved in regulation of metabolic processes and bone metabolism (Boeta-Lopez et al. 2018; Scheller et al. 2011).

In addition to the appropriate HLA combination, single nucleotide polymorphisms (SNPs) of cytokines and their receptors have been identified as factors which potentially affect the outcome after allogeneic HSCT (Balavarca et al. 2015; Mullally and Ritz 2007).

The gene of IL6 contains five SNPs (Jeon et al. 2010). The most extensively reported SNPs are the IL6-174 (G/C) polymorphism and the IL6-597 (G/A) polymorphism which are located in the promoter region. Both SNPs could influence the expression or repression of the IL6 gene and could affect the production of the cytokine (Terry et al. 2000; Fishman et al. 1998). Fishman et al. (1998) reported that healthy subjects possessing the IL6-174 G allele produce higher levels of IL6.This polymorphism has been shown to influence the risk of other diseases such as systemic-onset juvenile chronic arthritis (Fishman et al. 1998) and cardiovascular disease (Machal et al. 2014), and the outcome after renal transplantation (Marshall et al. 2001). High levels of IL6 correlate with the manifestation, the severity (Imamura et al. 1994; Steffen et al. 1996), and the prognosis (Lange et al. 1995) of acute GVHD.

We sought to investigate the impact of the IL6-174 and IL6-597 SNPs in both recipient and donor on the outcome after pediatric allogeneic HSCT. The IL6 polymorphisms could serve as prognostic factors for the occurrence of complications after transplantation and warrant the adjustment of prophylactic measures.

## Materials and methods

### Patients

The study population included 320 pediatric patients and their donors. All patients underwent transplantation for various hematological, genetic, or immunological diseases at the Department of Pediatrics, Jena University Hospital, Jena, Germany. All patients or their parents gave the informed consent for being included in the study. The protocol was authorized by the local ethic committee. We excluded patients who received more than one allogeneic HSCT and patients or donors for whom no SNP analysis was available. We obtained blood or bone marrow samples from remnant diagnostic samples. A detailed characterization of the study population is shown in Table 1.

**Table 1:** Characteristics of patients and donors (*n* = 320)

Characteristics	Total no. (%)
Median age of the patients (years)	10
Sex of patients	
Male	196 (61.2)
Female	124 (38.8)
Sex of donors	
Male	191 (59.7)
Female	129 (40.3)
Disease	
ALL	109 (34.1)
AML	73 (22.8)
CML	16 (5.0)
CMML	5 (1.6)
JMML	6 (1.9)
EWS/NBL/RMS	13 (4.1)
Genetic disease	42 (13.1)
Hemophagocytic lymphohistiocytosis	2 (0.6)
Hodgkin’s lymphoma	2 (0.6)
Myelodysplastic syndrome	31 (9.7)
Malignant histiocytosis/myelofibrosis	1 (0.3)
Non-Hodgkin lymphoma	9 (2.8)
Severe aplastic anemia	11 (3.4)
Conditioning regimen (based on)	
Total body irradiation	117 (36.6)
Chemotherapy	203 (63.4)
GVHD prophylaxis	
Cyclosporine A/methotrexate	194 (60.6)
Cyclosporine A	56 (17.5)
None	40 (12.5)
Others	30 (9.4)
aGVHD	
Total	145 (45.3)
Grade II–IV	88 (27.5)
Grade III–IV	31 (9.7)
cGVHD	
Total	52 (16.3)
Donor type	
HLA-matched unrelated	147 (45.9)
HLA-mismatched unrelated	41 (12.8)
HLA-identical related	94 (29.4)
HLA-haploidentical related	38 (11.9)
Cell type	
Bone marrow	224 (70.0)
Peripheral blood stem cells	94 (29.4)
Cord blood	2 (0.6)

*ALL* acute lymphoblastic leukemia, *AML* acute myeloid leukemia, *CML* chronic myeloid leukemia, *CMML* chronic myelomonocytic leukemia, *JMML* juvenile myelomonocytic leukemia, *EWS* Ewing sarcoma, *NBL* neuroblastoma, *RMS* rhabdomyosarcoma, *GVHD* graft-versus-host disease, *aGVHD* acute graft-versus-host disease, *cGVHD* chronic graft-versus-host disease

### Genetic analysis of interleukin 6 polymorphism

We used samples of peripheral blood, cord blood, and bone marrow aspirates to extract DNA. Mononuclear cells were purified by Ficoll–Hypaque (Sigma, St. Louis, MO, USA) and cryopreserved in liquid nitrogen at − 196 °C. DNA isolation was performed using the High Pure PCR Template Preparation Kit (Roche, Mannheim, Germany) according to the manufacturer’s instructions. Photometric DNA quantification at 260 nm and 280 nm was performed with the BioPhotometer plus (Eppendorf, Wesseling-Berzdorf, Germany).

We produced a mix containing 1 µl (10 ng/µl) DNA, 10 µl Genotyping Mastermix, 9.5 µl sterile aqua and 0.5 µl primer–probe mix. The samples were transferred by pipettes into 96-well optical reaction plates with barcodes. Additionally, at least five negative controls were used to verify quality assurance. For the absolute quantification process, the 7900HT Fast Real-Time PCR System (Applied Biosystems, Foster City, CA, USA) was used. At a temperature of 95 °C for 10 min, the enzymes in the sample plates were activated. Then, a total of 40 cycles followed on this process. The cycling conditions were 15 s at 92 °C for denaturation and 1 min at 60 °C for annealing and extension. Afterwards, an allelic discrimination post-read run was started. The PCR products were analyzed using the TaqMan SNP Genotyping Assay for genotyping the SNPs IL6-174 (rs1800795) and IL6-597 (rs1800797).

### Statistical analysis

The aim of our study was to identify an association between the IL6 polymorphisms 174 and 597 of recipients or donors and the outcome after allogeneic HSCT. We determined the following endpoints: overall survival (OS), event-free survival (EFS), relapse incidence (RI), transplant-related mortality (TRM), and the occurrence of acute GVHD and chronic GVHD.

The OS describes the time from transplantation to death, independent of the cause of death. We determined relapse, progression, death, and secondary malignancy as events for the EFS and it was investigated in the group of patients with leukemia. RI was defined as time to relapse or progression with death without relapse as competing risk. TRM describes the time to death without relapse or progression. The acute GVHD was diagnosed according to the classification by Przepiorka et al. (1995) and revised by the Mount Sinai Acute GVHD International Consortium, published by Harris et al. (2016). The National Institute of Health consensus established diagnostic criteria for chronic GVHD in 2005 (Filipovich et al. 2005) which were updated in 2014 (Jagasia et al. 2015) and in 2020 (Kitko et al. 2020).

OS and EFS were analyzed with the Kaplan–Meier method. To compare differences between the survival curves, we used the log-rank test. The calculations of RI, TRM, and GVHD were performed with the Gray test (Gray 1988). For multivariate analysis, we used cox regression to identify possible confounding variables such as age at time of transplantation, donor-recipient-gender match, source of stem cells, and HLA compatibility.

Statistical analysis was performed using the software IBM SPSS Statistics 27 and R Foundation. *P* values less than 0.05 were considered statistically significant.

## Results

### Frequency of interleukin 6 polymorphisms

The IL6-174 polymorphism was examined in 300 recipients and 295 donors. The IL6-597 polymorphism was analyzed in 299 recipients and 296 donors.

153 patients (47.8%) had the GC genotype of the IL6-174 SNP, 85 patients (26.6%) the GG genotype, and 62 patients (19.4%) the CC genotype. In 20 cases, genotype was not available. 144 donors (45.0%) were heterozygous for the IL6-174 GC genotype. 96 donors (30.0%) were homozygous for the GG genotype and 55 donors (17.2%) for the CC genotype. In 25 cases, DNA was not available for genotyping.

We found the IL6-597 GA, GG and AA genotypes in 149 recipients (46.6%), 91 recipients (28.4%) and 59 recipients (18.4%), respectively. We observed the GA genotype of the IL6-597 in 147 donors (45.9%), the GG genotype in 96 donors (30.0%) and the AA genotype in 53 donors (16.6%). In 21 recipients and 24 donors, DNA was not available for genotyping.

An overview of the genotypes’ frequencies is presented in Table 2.

**Table 2:** Genotype frequencies of the IL6-174 and IL6-597 SNPs in patients and donors

SNP	Patients (%)	Donors (%)
IL6-174		
Total	300 (93.8)	295 (92.2)
Genotype GG	85 (26.6)	96 (30.0)
Genotype GC	153 (47.8)	144 (45.0)
Genotype CC	62 (19.4)	55 (17.2)
IL6-597		
Total	299 (93.4)	296 (92.5)
Genotype GG	91 (28.4)	96 (30.0)
Genotype GA	149 (46.6)	147 (45.9)
Genotype AA	59 (18.4)	53 (16.6)

The distribution of genotypes was similar to those reported in the literature (Ambruzova et al. 2009; Mullighan et al. 2004; Gao et al. 2014).

Comparing the distribution of the SNPs revealed a strong linkage disequilibrium between IL6-174 GG/ IL6-597 GG, IL6-174 GC/IL6-597 GA, and IL6-174 CC/ IL6-597 AA in recipients and donors. Only in ten recipients (3.1%) and eight donors (2.5%), the above-mentioned genotypes were not linked. This result is in line with the findings of Muller-Steinhardt et al. (2004).

### Frequency of graft-versus-host disease

We observed acute GVHD in 145 patients (45.3%). 88 patients (27.5%) had a moderate to severe acute GVHD (grade II-IV) and 31 patients (9.7%) had a severe acute GVHD (grade III-IV). The median time to onset of acute GVHD was 28 days.

Chronic GVHD occurred in 52 patients which led to a chronic GVHD incidence of 16.3%. The median number of days between allogeneic HSCT and onset of chronic GVHD was 164 days.

### Genetic associations with acute graft-versus-host disease

**Fig. 1:**
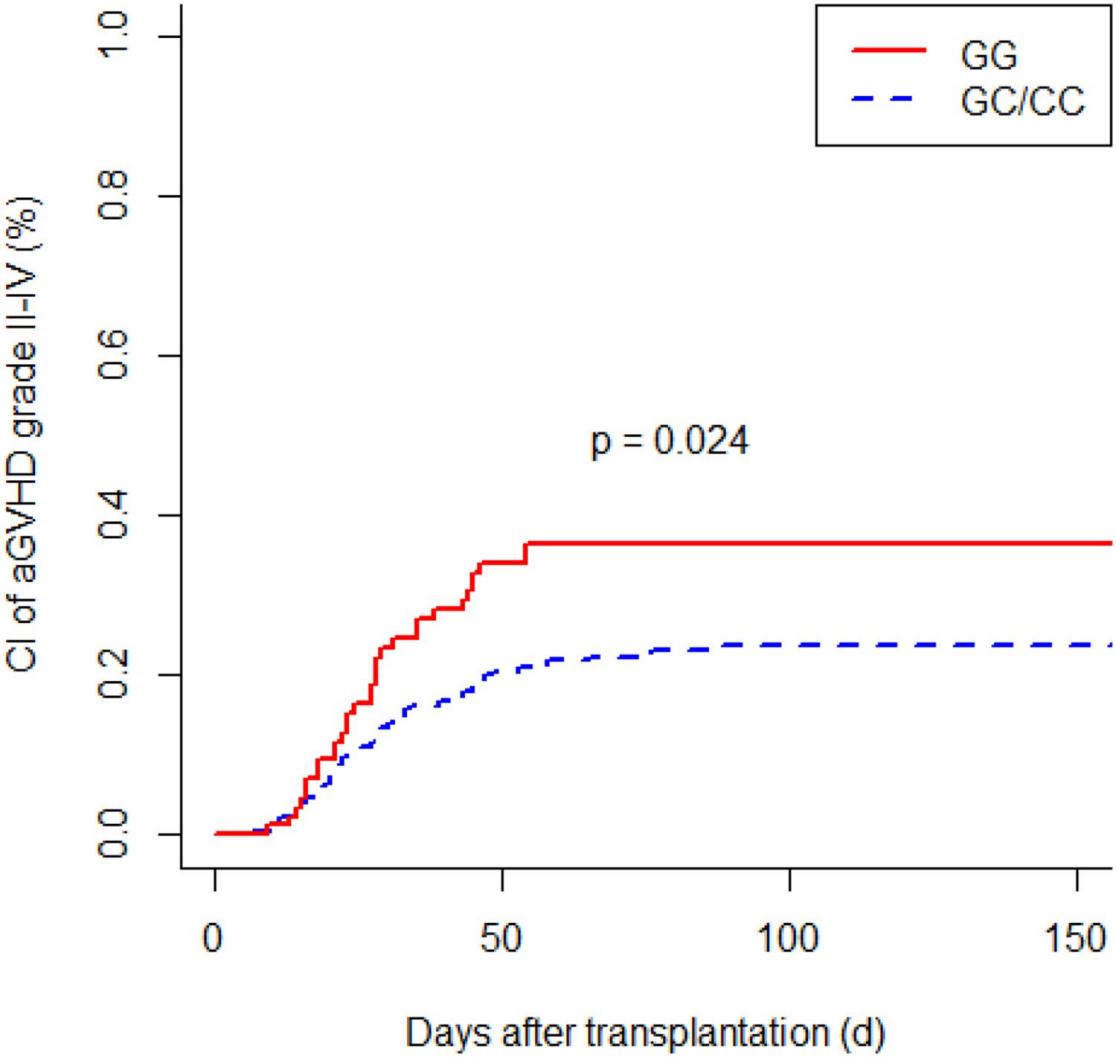
Cumulative incidence (CI) of acute graft-versus-host disease (aGVHD) (grade II–IV) according to recipients’ IL6-174 polymorphism

**Fig. 2:**
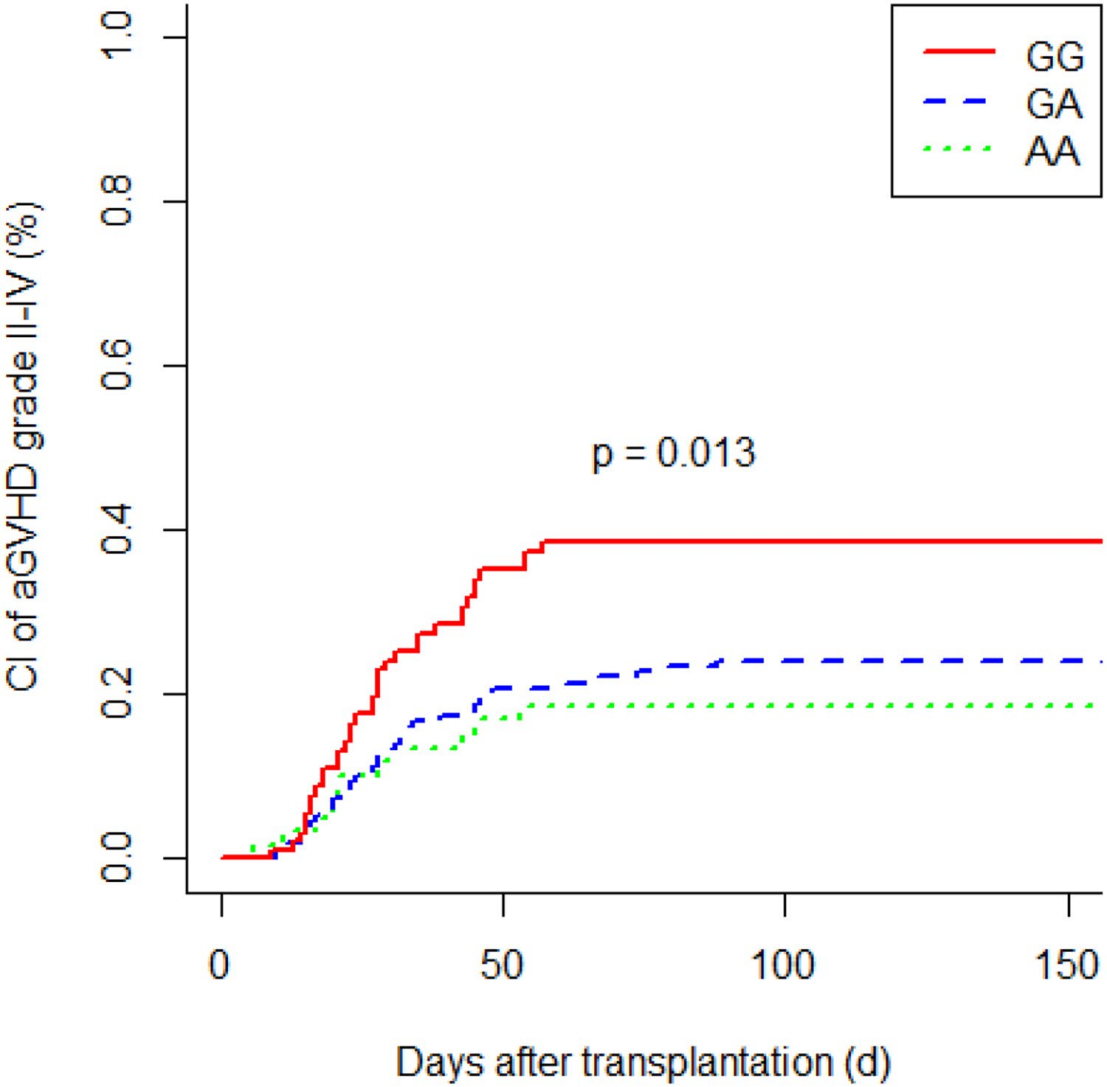
Cumulative incidence (CI) of acute graft-versus-host disease (aGVHD) (grade II–IV) according to recipients’ IL6-597 polymorphism

To investigate a possible association between recipients’ and donors’ IL6-174 and IL6-597 SNPs and the occurrence of acute GVHD, we compared the proportion of genotypes between recipients with clinically significant acute GVHD (grade II–IV) and severe acute GVHD (grade III–IV). We observed a significantly increased incidence of moderate to severe acute GVHD (grade II–IV) in recipients with IL6-174 GG genotype compared to those with IL6-174 GC/CC genotype (GG vs. GC/CC; *P* = 0.024; Fig. 1). Furthermore, patients with IL6-597 GG genotype developed grade II–IV acute GVHD more frequently than individuals with the GA or AA genotype (GG vs. GA vs. AA; *P* = 0.013; Fig. 2). Severe acute GVHD (grade III–IV) was significantly more frequent in patients with IL6-597 GG genotype than in those with IL6-597 GA/AA genotype (GG vs. GA/AA; *P* = 0.042). Analysis of the influence of donor SNPs on the occurrence of acute GVHD did not reveal any significant results. In summary, for both IL6 polymorphisms a recipient GG genotype was associated with an increased risk of acute GVHD. The donor genotype had no influence on the occurrence of acute GVHD.

### Genetic associations with chronic graft-versus-host disease

**Fig. 3:**
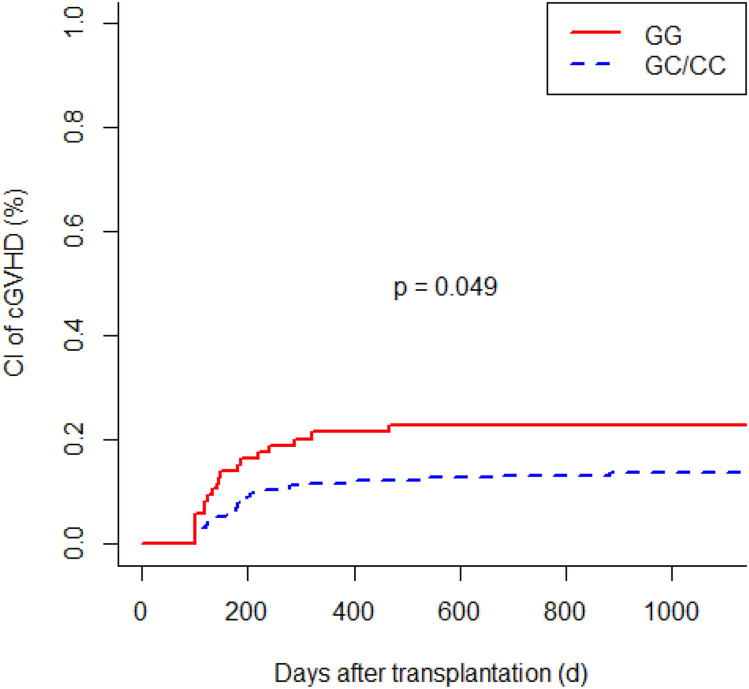
Cumulative incidence (CI) of chronic graft-versus-host disease (cGVHD) according to recipients’ IL6-174 polymorphism

**Fig. 4:**
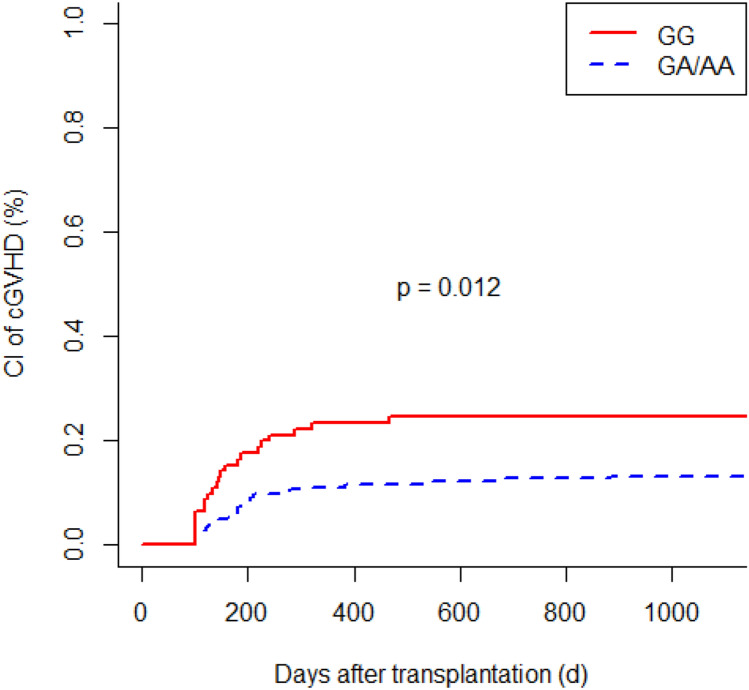
Cumulative incidence (CI) of chronic graft-versus-host disease (cGVHD) according to recipients’ IL6-597 polymorphism

The cytokine gene polymorphisms showed an association with the occurrence of chronic GVHD. Recipients with the IL6-174 GG genotype developed chronic GVHD more frequently than individuals with the C allele (GG vs. GC/CC; *P* = 0.049; Fig. 3). The chronic GVHD was present in 19 out of 85 patients (22.4%) with the GG genotype compared to 29 out of 215 patients (13.5%) with the GC/CC genotype. Similarly, we observed a significant association with an increased risk of chronic GVHD in recipients with IL6-597 GG genotype compared with patients with the other genotypes (GG vs. GA vs. AA; *P* = 0.043; GG vs. GA/AA; *P* = 0.012; Fig. 4). A total of 49 in the group of recipients’ IL6-597 SNP developed chronic GVHD: 22 of 91 patients (24.2%) with the GG genotype, 8 of 59 patients (13.6%) with the AA genotype and 19 of 149 patients (12.8%) with the GA genotype. In our study, the genotype of donors did not affect the incidence of chronic GVHD.

### Other endpoints

In addition to the occurrence of acute GVHD and chronic GVHD, we investigated the influence of the IL6-174 and IL6-597 polymorphisms on OS, EFS, RI, and TRM. The two SNPs had no significant impact on OS of the patients. We found no association between the polymorphisms and the EFS. There was no relationship between RI or TRM and the IL6-174 and IL6-597 SNPs.

### Multivariate analysis

Multivariate analysis was performed to examine the significance of clinical factors potentially influencing the occurrence of acute GVHD and chronic GVHD. To evaluate whether IL6-174 and IL6-597 SNPs are independent prognostic factors, we included following possible confounding variables: age at time of transplantation, donor-recipient-gender match, source of stem cells, and HLA compatibility.

In comparison with the above-mentioned variables, the recipients’ IL6-174 polymorphism remained to be an independent significant risk factor for the occurrence of acute GVHD (*P* = 0.030) and chronic GVHD (*P* = 0.045). The results of recipients’ IL6-174 polymorphism and acute GVHD and chronic GVHD are presented in Table 3. Furthermore, the IL6-597 SNP of the recipients turned out to be an independent significant prognostic factor for acute GVHD (*P* = 0.007) and chronic GVHD (*P* = 0.015). The results of recipients’ IL6-597 polymorphism and acute GVHD and chronic GVHD are shown in Table 4.

**Table 3:** Multivariate analysis of recipients’ IL6-174 SNP

Variable	aGVHD		cGVHD	
	HR (95% CI)	*P*	HR (95% CI)	*P*
Recipient IL6-174 (GG vs. GC/CC)	1.669 (1.051–2.65)	0.030	1.880 (1.014–3.49)	0.045
Gender match	0.989 (0.635–1.54)	0.960	0.997 (0.547–1.82)	0.990
HLA match	0.658 (0.403–1.07)	0.094	0.658 (0.344–1.26)	0.200
Stem cell source (PBSC vs. BM)	0.670 (0.394–1.14)	0.140	0.469 (0.219–1.01)	0.052
Age at time of transplantation	1.031 (0.994–1.07)	0.100	1.068 (1.023–1.12)	0.003

*HR* hazard ratio, *CI* confidence interval, *aGVHD* acute graft-versus-host disease, *cGVHD* chronic graft-versus-host disease, *PBSC* peripheral blood stem cells, *BM* bone marrow

**Table 4:** Multivariate analysis of recipients’ IL6-597 SNP

Variable	aGVHD		cGVHD	
	HR (95% CI)	*P*	HR (95% CI)	*P*
Recipient IL6-597 (GG vs. GA/AA)	1.863 (1.187–2.93)	0.007	2.053 (1.147–3.67)	0.015
Gender match	0.967 (0.624–1.50)	0.880	1.003 (0.562–1.79)	0.990
HLA match	0.661 (0.405–1.08)	0.098	0.696 (0.362–1.34)	0.280
Stem cell source (PBSC vs. BM)	0.689 (0.404–1.17)	0.170	0.472 (0.220–1.01)	0.054
Age at time of transplantation	1.030 (0.993–1.07)	0.110	1.068 (1.023–1.11)	0.003

*HR* hazard ratio, *CI* confidence interval, *aGVHD* acute graft-versus-host disease, *cGVHD* chronic graft-versus-host disease, *PBSC* peripheral blood stem cells, *BM* bone marrow

In addition to these findings, age at time of transplantation was a significant risk factor for the occurrence of chronic GVHD (*P* = 0.003). This is in line with the reported observation that older patient age is associated with an increased development of chronic GVHD (Bernaudin et al. 2020; Shreberk-Hassidim et al. 2018).

## Discussion

Success of allogeneic HSCT is still compromised by the occurrence of various complications (Ambruzova et al. 2009). New approaches investigated non-HLA-associated factors, in particular genetic variants of cytokines, as possible risk factors in allogeneic HSCT (Balavarca et al. 2015; Mullally and Ritz 2007). IL6 is an important cytokine which plays a crucial role in inflammatory processes and infection response (Scheller et al. 2011). Our study investigated the impact of the IL6-174 and IL6-597 SNPs of recipient and donor on the outcome after allogeneic HSCT in childhood.

Our findings are in partial agreement with previously published data. While some studies reported an association between IL6 polymorphisms of donor (Balavarca et al. 2015; Cavet et al. 2001; Chien et al. 2012), we did not observe a significant connection. This inconsistence might be explained by the differences in clinical practice such as different conditioning regimens, GVHD prophylaxes or ethnic background. Many studies investigated the association between the polymorphisms and the outcome only in related or sibling donor-recipient pairs (Cavet et al. 2001; Karabon et al. 2005; Ambruzova et al. 2009). On the other hand, we performed our analysis in an exclusively pediatric patient population whereas other publications included data from adult patients (Choi et al. 2012).

We observed that patients with the IL6-174 GG genotype developed acute GVHD (grade II–IV) more frequently than individuals with the C allele. While our data are in agreement with most previous studies which suggest a relationship between the IL6-174 GG genotype of the recipients and an increased risk for development of acute GVHD (Ambruzova et al. 2009; Cavet et al. 2001; Dickinson and Charron 2005; Dukat-Mazurek et al. 2017), other authors did not observe an association between IL6-174 SNP and acute GVHD (Lin et al. 2003; Mullighan et al. 2004; Tvedt et al. 2018). To our knowledge, only one study focused on a detailed analysis of the impact of IL6-597 SNP on the outcome of allogeneic HSCT. Ambruzova et al. (2009) described that acute GVHD tended to be more frequent in patients with IL6-597 GG genotype. We found a significant association between recipients’ IL6-597 GG genotype and the occurrence of acute GVHD.

The IL6-174 G allele is associated with a higher serum level of IL6 (Fishman et al. 1998). The IL6-174 and IL6-597 SNPs are located in the promoter region and can influence the expression or repression of this gene. Terry et al. (2000) reported that the IL6-174 polymorphism does not act independently of other polymorphisms. Regulation of transcription is a result of the combination of different polymorphisms (Terry et al. 2000). One possible explanation for various effects on expression is that the polymorphisms can influence the affinity for transcription activators or repressors. Alternatively, different allele combinations can bind different transcription factors, each factor with a special effect on gene expression (Kelberman et al. 2004). For example, some factors can bind when IL6-597 G allele and IL6-174 G allele are combined which results in an increased transcription. Other factors can bind when IL6-597 A allele and IL6-174 C allele are combined which results in a decreased transcription (Muller-Steinhardt et al. 2004).

IL6 is also produced by non-hematopoietic cells such as adipocytes, endothelial cells, fibroblasts, mesangial cells, and vascular smooth muscle (Terry et al. 2000; Marshall et al. 2001). These cells are not affected by the transplantation. Consequently, the IL6 production remains dependent on the genotype of the recipient after allogeneic HSCT.

In the pathophysiology of acute GVHD, the pretransplant conditioning leads to the release of pro-inflammatory cytokines. This release is dependent on the IL6 genotype of patients. Some patients are predisposed to high serum levels of these cytokines and thus predisposed to the development of acute GVHD (Dickinson and Charron 2005).

There are conflicting data regarding the IL6-174 gene polymorphism and the risk of chronic GVHD. Socie et al. (2001), Dickinson et al. (2001) and Cavet et al. (2001) found an association between the recipients’ IL6-174 GG genotype and an increased incidence of chronic GVHD. On the contrary, other authors did not describe a relationship (Ambruzova et al. 2009; Lin et al. 2003). We observed a significant association between the IL6-174 GG genotype of recipients and the occurrence of chronic GVHD. In the study of Ambruzova et al. (2009), IL6-597 SNP did not affect the incidence of chronic GVHD. Contrary to this, we found a significant association between recipients’ IL6-597 GG genotype and the risk of chronic GVHD. Compared with the knowledge about acute GVHD, the pathophysiology of chronic GVHD is poorly defined and there are many explanation attempts. Lee et al. (2003) regarded chronic GVHD as a process of cytokine dysregulation. High levels of IL6 could be associated with more severe forms of chronic GVHD.

We did not find any significant relationships between OS, EFS, RI, TRM and the IL6-174 and IL6-597 polymorphisms of recipient and donor. Ambruzova et al. (2009) and Tvedt et al. (2018) described an association between the IL6-174 polymorphism and TRM. Ambruzova et al. (2009) and Balavarca et al. (2015) observed a relationship between OS and IL6-174 G allele.

In multivariate analysis, we confirmed that IL6-174 and IL6-597 SNPs are independent significant risk factors for the occurrence of acute GVHD and chronic GVHD. In addition, older age at time of transplantation turned out to be a significant risk factor for the development of chronic GVHD.

We conclude that IL6-174 and IL6-597 SNPs of pediatric patients were significant risk factors for the development of acute GVHD and chronic GVHD. For both IL6 polymorphisms, recipients with GG genotype developed acute GVHD and chronic GVHD more frequently than individuals with C or A allele. The IL6-174 G allele is associated with a higher serum level of IL6 (Fishman et al. 1998) resulting in an increased inflammatory response and a higher risk to develop acute GVHD and chronic GVHD. The IL6 polymorphisms are prognostic factors for the occurrence of GVHD after transplantation. Our results can contribute to an intensification of GVHD prophylaxis and can justify a new treatment option such as IL6-receptor blocking with tocilizumab in patients with severe GVHD (Kattner et al. 2020). Our study adds new information to the existing body of knowledge about non-HLA immunogenetics to identify allogeneic HSCT recipients at high risk for acute GVHD and chronic GVHD. Further research in larger and more homogenous cohorts are necessary to confirm our results.
